# Quantitative factors proposed to influence the prevalence of canine tick-borne disease agents in the United States

**DOI:** 10.1186/1756-3305-7-417

**Published:** 2014-09-04

**Authors:** Roger W Stich, Byron L Blagburn, Dwight D Bowman, Christopher Carpenter, M Roberto Cortinas, Sidney A Ewing, Desmond Foley, Janet E Foley, Holly Gaff, Graham J Hickling, R Ryan Lash, Susan E Little, Catherine Lund, Robert Lund, Thomas N Mather, Glen R Needham, William L Nicholson, Julia Sharp, Andrea Varela-Stokes, Dongmei Wang

**Affiliations:** Department of Veterinary Pathobiology, University of Missouri, Columbia, MO USA; Department of Pathobiology, Auburn University, Auburn, AL USA; College of Veterinary Medicine, Cornell University, Ithaca, NY USA; Companion Animal Parasite Council, Salem, OR USA; School of Veterinary Medicine and Biomedical Sciences, University of Nebraska, Lincoln, NE USA; Department of Veterinary Pathobiology, Oklahoma State University, Stillwater, OK USA; Walter Reed Biosystematics Unit, National Museum of Natural History, Washington, DC USA; Department of Medicine and Epidemiology, University of California, Davis, CA USA; Department of Biological Sciences, Old Dominion University, Norfolk, VA USA; Department of Forestry, Wildlife and Fisheries, University of Tennessee, Knoxville, TN USA; Department of Geography, University of Georgia, Athens, GA USA; City Kitty Veterinary Care for Cats, Providence, RI USA; Department of Mathematical Sciences, Clemson University, Clemson, SC USA; Center for Vector-Borne Disease, University of Rhode Island, Kingston, RI USA; Department of Entomology, The Ohio State University, Columbus, OH USA; Centers for Disease Control and Prevention, Atlanta, GA USA; Department of Basic Sciences, College of Veterinary Medicine, Mississippi State University, Kragujevac, Mississippi State, MS USA

**Keywords:** *Anaplasma*, *Ehrlichia*, *Borrelia burgdorferi*, Tick-borne infections, Prevalence map factors, Ticks, Ixodidae, Prostriata, Metastriata

## Abstract

The Companion Animal Parasite Council hosted a meeting to identify quantifiable factors that can influence the prevalence of tick-borne disease agents among dogs in North America. This report summarizes the approach used and the factors identified for further analysis with mathematical models of canine exposure to tick-borne pathogens.

## Background

Dogs in the United States (USA) are hosts to a diverse range of ixodid ticks and can become infected with many of the pathogens transmitted by these vectors. Advances in diagnostic test and recording technologies have led to the creation of a monthly dataset containing county-by-county canine test results from across the USA. The Companion Animal Parasite Council (CAPC) has assembled large datasets of such results from commercial laboratories that provide diagnostic tests for canine exposure to *Borrelia burgdorferi*, *Ehrlichia* spp. and *Anaplasma* spp. [1]. These monthly, county-level CAPC prevalence maps generated interest in the utility of the datasets for assessing seroprevalence norms, forecasting future seroprevalence rates and for identifying trends in canine exposure to this array of tick-borne disease agents. A group of vector ecologists, parasitologists, other biologists and statistical modelers met in Atlanta, GA (June 9–10, 2012) to identify factors that could enhance the accuracy of these predictive models. This report narrates the results of the meeting.

Canine diagnostic test results for exposure to tick-borne pathogens, including *B. burgdorferi*, *Ehrlichia* spp. and *Anaplasma* spp., are of significant interest, not only because canine health is important to pet owners and veterinarians, but also because of the public health importance of many of these infectious disease agents. These tick-borne pathogens are transmitted by two phylogenetically distinct groups of ixodid ticks. Members of the ixodid subfamily Prostriata (*Ixodes* spp.) transmit agents of granulocytic anaplasmosis (*Anaplasma phagocytophilum*) and Lyme borreliosis (*B. burgdoferi*) and are likely to include vectors of a more recently described *Ehrlichia muris*-like agent in the USA. Members of the subfamily Metastriata (*e.g.,* the genera *Amblyomma*, *Dermacentor* and *Rhipicephalus*) transmit agents of canine and human ehrlichiosis (*e.g., E. canis, E. chaffeensis* and *E. ewingii*), canine anaplasmosis (*A. platys*) and spotted-fever group rickettsiosis (*i.e., Rickettsia rickettsii, R. conorii* and related *Rickettsia* spp.).

Large datasets have been assembled from reports of diagnostic test results for canine exposure to *B. burgdorferi*, *Anaplasma* spp. and *Ehrlichia* spp. in the USA. For example, from reports submitted nationwide from 2010–2012, 509,195 (7.2%) of 6,996,197 canine samples were seropositive for *B. burgdorferi*, 270,168 (4.4%) of 6,192,268 samples were seropositive for *Anaplasma*, and 111,673 (1.1%) of 6,994,683 samples were seropositive for *Ehrlichia* [2]. A previous national survey, spanning 2001–2007, reported results from 982,336 diagnostic tests for canine exposure to *B. burgdorferi* and *Ehrlichia* spp., and 479,640 tests for canine antibodies to *Anaplasma* spp., with 5.1%, 0.6% and 4.7% of these samples testing seropositive for *B. burgdorferi, Ehrlichia* and *Anaplasma*, respectively [3]. Interestingly, when the canine seroprevalence of *B. burgdorferi* in the 2001–2007 study was compared to the subsequent prevalence of human Lyme disease, the most commonly reported human vector-borne illness in the USA, canine seroprevalence of *B. burgdorferi* ≥5.1% was predictive of emergent human Lyme disease in low-incidence counties; a low canine seroprevalence (≤1.0%) was associated with minimal risk for emergent human Lyme disease [4]. A subsequent report, however, underscored the importance of other variables, such as the distribution of competent vector species, for accurate interpretation of these canine diagnostic test data [5].

The overall objective of this CAPC-sponsored workshop was to identify factors that are likely to influence the seroprevalence of canine exposure to tick-borne disease agents in the USA, specifically focusing on the factors and the pathogens for which sufficient data are available, so that these factors could be evaluated for incorporation in mathematical models designed to monitor and to predict spatial and temporal seroprevalence patterns. These preliminary factors provided statisticians some of the critical information needed to begin their model-building procedures.

### Approach

**Figure 1:**
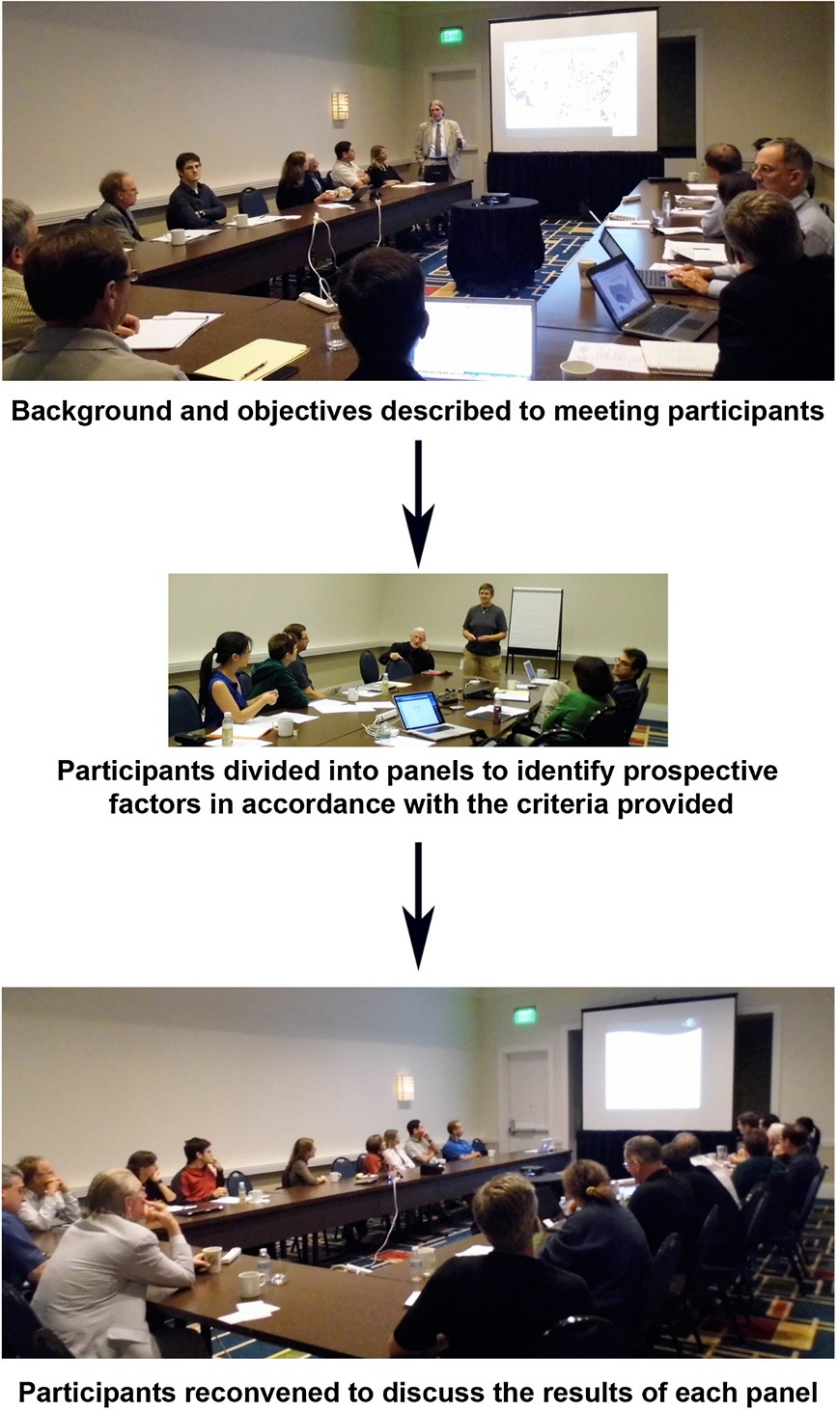
**Approach to rational identification of quantitative factors proposed to influence the exposure of dogs to vector-borne pathogens.** Background information, meeting objectives and guidelines were presented to participants before they were divided into three separate panels, according to vector taxa, for mosquitoes, prostriate ticks and metastriate ticks. Each panel was asked to identify, discuss and to rank candidate factors for evaluation with statistical prevalence models of pathogens transmitted to US dogs by each vector taxon. All of the participants were subsequently reconvened for further discussion and refinement of the results from each panel.

Two teams of researchers, from various areas of tick and tick-borne pathogen biology, were assembled and tasked with rational identification of factors thought to be relevant to the canine seroprevalence of pathogens transmitted by prostriate (eight team members) or metastriate (seven team members) ticks (Figure 1). Members of each team were selected based upon diverse areas of expertise in tick biology, tick-borne disease, vector ecology or statistics. Each panel was asked to identify and to rank ten key factors that they considered most likely to affect pathogen seroprevalence, and these preliminary factors were then presented to all of the meeting participants for further discussion. It was understood that the relevance of these factors would be subsequently assessed with mathematical models, and that these models would be adjusted with data that continue to be generated. Thus, the utility of different factors would be continually assessed as the mathematical models are refined over time.

The working groups for both ixodid subfamilies began by discussing variables categorized as (1) vector, (2) host, (3) abiotic, (4) habitat or (5) social. Both groups independently identified numerous factors. The majority of factors were thought to be associated with canine exposure to pathogens vectored by either ixodid subfamily; however, several factors specifically associated with the different ixodid subfamilies also emerged. Variables were also discussed for which there is little or inconsistent supporting data, but these factors could become useful if the data became available. However, in accordance with the workshop objectives, factors for which sufficient data are currently available were chosen for ranking by consensus of each working group.

The variables independently identified by each panel were categorized into the five groups previously indicated (i.e., vector, host, abiotic, habitat and social). Factors regarding exposure to infectious agents transmitted by prostriate ticks were heavily influenced by the preponderance of research on the phenology of *Ixodes scapularis* and *I. pacificus*, which are considered the primary vectors of *B. burgdorferi* and *A. phagocytophilum* in North America (Table 1). For metastriate-borne pathogens, host biology and human behavior were second only to vector distribution with regard to factors considered likely to influence seroprevalence (Table 2). Brief explanations and comments regarding these factors are described below.

**Table 1:** **Factors initially considered as potential contributors to canine prevalence of disease agents transmitted by** ***Ixodes scapularis*** **and** ***I. pacificus***

Vector factors	
	Distribution
	Abundance
	% Infected
	Canine contact
	Local phenology
	Tolerance to temperature and humidity
	Activity
	Focus on adults as primary vector to dogs
	Host seeking behavior
	Host contact
	Feeding preferences and opportunities
	Deer population drives tick abundance
	Small mammal population drives infection prevalence
	Lack of lizards
	Diversity/dilution effect
	Tick encounters
	Questing behavior versus relative humidity
	Peridomestic encounters – access to areas
	Urbanization/Rate of development
	Infection status (decreased survival versus increased cold tolerance)
**Host factors**	
	Presence and abundance (deer, small mammals, lizards)
	Dilution effect/host diversity
	Habitat availability and quality
	Mast crop as a surrogate for host reproduction/fitness
	Migratory bird patterns
	Reproductive capacity and timing of vertebrate host reproduction
	Population control programs in place locally
	Abiotic host survival factors
	Temperature, water availability, substrate/nesting material, snow cover
	Feeding preferences
	Herd immunity of reservoir host populations
	Hunting pressure/success
	Number of deer killed per county – harvest rates
	Hunting license versus hunting harvest – how active hunting is for that area
	Hunting limits due to development
**Abiotic factors**	
	Snow cover – depth, duration
	Miles of roads – neighborhood roads (non-interstate/parkway/highway), trails
	Soil type – clay versus sand in Northeastern USA
	Hydrological features
	*I. scapularis*
	Maximum temperature, warmest month
	Annual precipitation
	*I. pacificus*
	Minimum temperature, coldest month
	Daily temperature (high, low and average)
	Relative humidity (average, high, low, duration)
**Habitat factors**	
	Land cover classification
	Urbanization in 3 categories – low, medium, high
	Rate of change
	Forest cover
	Land cover classification (categorical), % canopy cover, NDVI, EVI (canopy structure)
	Crop phenology – maximum greening, minimum greening – when greening is happening
	Supervised vs unsupervised satellite imagery, derived data not currently off the shelf
	Forest type, forest fragmentation, forest edge length, forest composition, forest connectivity
	Forest fragments within X distance of road or urban area, close to population centers
	Understory- could be modeled but is not measured
	Detritus layers/leaf litter
	Targeted for future research but perhaps not currently available dataset
	Soil maps/soil types
	World harmonized soil database
	Classification scheme
	Proximity to rivers/drainage areas
	Proximity to coast
	Rain shadows
	Rivers and streams
	Attract hosts
	Serve as corridors
	Provide humidity
	Aspect/slope/topo index – derived from digital elevation models, available from hydro dataset
	More nymphal deer ticks on north- and east-facing slopes
	Effective distance – more ticks on uphill side of a payout
	Ticks associated with east-facing woodland edges that slope down to water
	Fire
	Eliminates leaf litter, changes food availability, changes microclimate
	Depending on timing, burn can increase number of infected ticks, so fewer ticks but higher infection rate
	Park boundaries – proximity to parks
**Social factors**	
	Human population centers
	Dog ownership, dog lifestyle
	Hunting styles that use dogs
	Breed of dog
	Dog ownership increase – by region
	More homes in tick habitat – demographic factors
	Deer/vehicle collisions – deer crossing signs
	Acaricide use/quality of care for dogs
	Average household income
	Presence of clinics, proximity to clinics, number of vet clinics in an area, size of clinics
	Cultural – forest foraging (mushroom hunting in Missouri)
	Internet use
	Social media
	Smartphone use
	Education level
	Population density
	Housing type (average lot size, median home price, age of house unit, census tract size)

**Table 2:** **Factors discussed as potential contributors to seroprevalence of metastriate tick-borne pathogens among dogs in the USA**

Vector factors	
	Biology
	Competence (different transmission scenarios)
	Host preference
	Persistence and interhost transfer of male ticks
	Host seeking behavior (hunt, ambush)
	Population dynamics
	Distribution (established, intermittent or absent)
	Relative abundance (species and stages)
	Seasonality
	Different stages
	Stage overlap
**Host factors**	
	Principal host(s) of different tick stages
	Susceptibility to pathogen
	Distribution
	Density-Dynamic
	Ecologic diversity (dilution effect)
	Shannon-Weaver Index
	Tick-permissive, non-reservoir hosts
	Behavior
	Host grooming
	Gregariousness
	Host species
	Home Range
	Migration, dispersal
	Anthropogenic translocation
	Hosts permissive for pathogen
	Persistence in reservoir
	Prevalence of infection
	Density
	Other transmission routes
	Life cycle/age distribution
	Immune response
	Amplification vs. reservoir
	Domestic
	Indoor/outdoor
	Rural/urban
	Relocation
	Sylvatic vs. Suburban
	Opportunistic or natural infection
**Abiotic factors**	
	Humidity
	Maximum, minimum and average
	Temperature
	Maximum, minimum and average
	Degree-day
	Soil temperature
	Photoperiod
	Seasonal precipitation
	El Niño effect
	Snow and other ground cover
	Catastrophic disturbance
	Fire
	Hurricane
	Wind
	Altitude
**Habitat factors**	
	Macrohabitat
	Vegetation (density, type and fragmentation)
	Elevation
	Location of water sources
	Rainfall
	Microhabitat
	Soil type
	LIDAR data
	Land use
**Social factors**	
	Land use
	Indoor versus outdoor dogs
	Dog use (*e.g.,* hunting)
	Canine husbandry
	Use of tick preventives
	Nuisance permits
	Housekeeping
	Animal welfare violations
	Socioeconomics
	Average household income
	Human population
	Large-scale economic factors
	Recreation
	Hunting
	Parks (rural and urban)
	Pets per household

### Vector factors

#### Distribution

The geographic distribution of prostriate ticks was focused on the *Ixodes* spp. thought to most commonly feed on dogs (and people) in the USA: *I. scapularis* and *I. pacificus*. Metastriate ticks considered as pathogen vectors (e.g., of *Ehrlichia* spp. and *A. platys*) included, in alphabetical order, *A. americanum, A. maculatum, D. andersoni, D. variabilis* and *R. sanguineus*. The general distributions of these ticks are relatively well documented in the literature and via voucher specimens in the USA. However, the spatial resolutions of these data vary in different regions, and defining the minimum useful scale can be complicated by discontinuous geographic distributions of tick populations in a given area.

#### Abundance

Defining permanent values of tick abundance levels is problematic, because tick population levels within a given area are temporally and spatially variable and can change rapidly. Tick abundance depends on host abundance and availability, relative humidity, precipitation and temperature, and can reflect conditions from previous years when immature tick stages or prior generations were active.

#### Activity

Activity is indicative of questing behavior, host-seeking behavior, host contact and the feeding preferences of different developmental stages. The presence of ticks in an area is not alone indicative of activity. For example, tick activity will depend on temperature, precipitation, relative humidity and photoperiod.

### Host factors

#### Deer

The deer population is a major driver of abundance for certain ticks, such as *I. scapularis, I. pacificus* and *A. americanum*. Deer are also a reservoir of *E. chaffeensis* and could be involved in the maintenance of *E. ewingii*.

### Small mammals

Rodents are an important component of the ecologies of several tick species and some tick-borne infectious agents. Immature stages of several tick species acquire blood meals from small vertebrate hosts. Several tick-borne infectious agents, such as *B. burgdorferi*, *A. phagocytophilum* and *R. rickettsii* are adapted to rodent reservoir hosts.

#### Lizards

Small vertebrates such as lizards, which are permissive hosts for immature tick stages but are not definitively documented reservoirs of the pathogens under consideration, could dampen transmission of disease agents that are adapted to rodent reservoirs. Conversely, removal of lizards reportedly reduced nymphal tick numbers from an environment but did not affect the percentage of *B. burgdorferi*-positive ticks, suggesting that increased numbers of lizard hosts might actually increase the risk of pathogen transmission by serving to increase the overall number of ticks in a given area [6].

#### Migratory bird patterns

Migratory birds can introduce some tick species to new areas [7]. However, ticks that feed on dogs and that are dispersed by birds in the USA may be incapable of maintaining an active population cycle in the absence of larger vertebrate hosts (*e.g.,* white-tailed deer).

#### Abiotic factors

Different tick species and their natural hosts can be adapted to various environments that are influenced by abiotic factors such as precipitation, temperature, relative humidity and soil composition.

#### Habitat factors

Factors that influence the life cycles of ticks and their vertebrate hosts include vegetation, urbanization, land use in non-urban settings and detritus layers.

#### Social factors

Human behavior and population characteristics influence the exposure of dogs to ticks. These include access to preventive care, recreation, socioeconomic status, income, pathogen reservoir control, vector-amplification host control and news media coverage.

### Unquantified variables

A number of variables were discussed for which comprehensive, nationwide data did not seem currently available. These variables included vector infection rates, detailed reservoir infection rates, vector abundances, vector efficiency indices, vector survival, vectorial capacities, temperature-dependent development rates of vectors (natural temperature regimes), total number of dogs (by county or zip code) and tick control product sales in each geographic region. Local data may be available for some of these variables in certain areas, but national datasets were not available at the time of this meeting.

### Mathematical modeling

Each expert panel was asked to prioritize 10 factors most expected to drive a reliable mathematical predictive model. These lists, summarized in Tables 3 and 4, shared several common abiotic and habitat factors. Several other factors were specific to seroprevalence of the pathogens transmitted by *Ixodes* spp., *R. sanguineus* or the other metastriate ticks that were considered. For example, while deer populations and vegetation were considered important factors that affect the majority of these tick populations, social factors were given the highest priority for predicting the seroprevalence of agents transmitted by the brown dog tick, *R. sanguineus* (Table 4).

**Table 3:** **Ranked factors identified for canine seroprevalence models of infections transmitted by** ***Ixodes*** **spp. in the USA**

1.	Forest cover/NDVI or EVI^a^
2.	Relative humidity
3.	Annual precipitation (including snow cover)^a^
4.	Human population density^a^
5.	Deer/vehicle collisions^a^
6.	Topography/altitude/aspect
6.	Temperature – max warmest, min coldest^a^
7.	Proximity of forest to impervious surfaces or roads/built environment
8.	Human case distribution
8.	Distribution/abundance of *I. scapularis* and *I. pacificus* ^a^
9.	Household income^a^
10.	Forest fragmentation index^a^

^a^Similar variables also ranked by the metastriate-borne pathogen panel.

**Table 4:** **Ranked factors for preliminary models of metastriate tick-borne pathogen prevalence among dogs in the USA**

Majority of the metastriata:	
1.	Vector distribution (established, intermittent or absent)^a^
2.	Maximum, minimum and average temperature^b^
3.	Amount of precipitation^a^
4.	LiDAR (up to 6 layers)
5.	GAP/categorical analysis of vegetation^a^
6.	Reservoir host densities^a^
7.	Human population (census)^a,b^
8.	Median household income^a,b^
9.	Fragmentation of vegetation^b^
10.	Degree-days
11.	Seasonal precipitation (snow cover)^a^
***R. sanguineus*** **:**	
1.	Median household income ^a,b^
2.	Registered dog breeders (kennels, puppy mills, *etc*.)
3.	Human population (census)^a,b^
4.	Tick preventive sales
5.	Animal welfare violations
6.	Latitude

^a^Variables also ranked by the prostriate-borne pathogen panel.

^b^Variables shared among all ixodid ticks considered for this report.

The prevalence data at the foundation of this predictive model is largely based on serodiagnostic tests. Although seropositivity is reflective of past exposure, it does not demonstrate recent or active infections. Repeatedly seropositive samples from the same dogs at different times are also to be occasionally expected, because some dogs may have tested seropositive in previous tests and because some tests are conducted to monitor host responses to treatment. Travel histories and certainties of the individual test results are currently unavailable for the dogs reported in this dataset.

An analogous project for mathematical modeling of the prevalence of canine heartworm was simultaneously undertaken by CAPC [8, 9], and each prioritized factor identified by the expert panel had significant predictive power with ≥95% confidence. Overall, the model explained 60%-70% of variability in the CAPC county-by-county dataset from 2011–2013. Similarly, preliminary analysis of canine seroprevalence of *Anaplasma* spp. indicated that temperature, precipitation, relative humidity, population density, median household income, forestation coverage, elevation and deer/vehicle strike rates were significant with ≥95% confidence, and that the total proportion of variability explained in the 2011–2013 data is around 60-70% [10]. Thus, the prevalence of heartworm and seroprevelance of *Anaplasma* among dogs appear amenable to quantification that could facilitate monitoring for outbreaks, remediation of vector abundance or for forecasting future seroprevalence levels.

Attempts to fit the seroprevalence of *B. burgdorferi* and of *Ehrlichia* spp. among dogs are also underway, with mixed results. The spatial seroprevalence of *B. burgdorferi* among dogs has been similar to and appears to be as quantifiable as that of *Anaplasma* spp. Conversely, the canine seroprevalence of *Ehrlichia* spp. appears to be highly variable, with some neighboring areas reporting antipodal seroprevalence rates that could be reflective of vector ecology or social factors. Future work will address these issues.

## Conclusions

This meeting brought together a range of junior and senior scientists engaged in various aspects of research in the biology of ticks and tick-borne infections. The specific objectives were to identify and to prioritize quantifiable factors expected to contribute to canine exposure to organisms transmitted by the two major subfamilies of ixodid ticks. The two panels ranked 12 and 17 factors associated with prostriate and metastriate ixodid ticks, respectively. Eight of these factors were independently prioritized by both panels; four of 12 factors were unique to prostriate-vectored agents, two of 11 factors were unique to metastriate-vectored agents transmitted by ticks other than *R. sanguineus*, and four of six factors were unique to agents vectored by *R. sanguineus*. The next phase of this project will move from rational identification of perceived factors to statistical assessment of factors for predictive power. Forecasting issues will also be explored.
